# Reconstruction of Severely Atrophied Mandible and Simultaneous Implant Insertion with an Inverted Sandwich Technique

**DOI:** 10.1155/2023/2973079

**Published:** 2023-01-04

**Authors:** Milad Etemadi Sh, Alireza Tamizifar, Fatemeh Aghajani, Mahshad Saei

**Affiliations:** ^1^Department of Oral and Maxillofacial Surgery, Dental Implants Research Center, Dental Research Institute, School of Dentistry, Isfahan University of Medical Sciences, Isfahan, Iran; ^2^Oral and Maxillofacial Surgery, Shahrekord University of Medical Sciences, Shahrekord, Iran; ^3^School of Dentistry, Student Research Committee, Islamic Azad University, Isfahan (Khorasgan) Branch, Isfahan, Iran

## Abstract

**Objectives:**

To reconstruct and rehabilitate a severely atrophied mandible. *Case presentation.* A 40-year-old female patient with the chief complaint of denture instability was admitted to our care. In our opinion, the optimum reconstructive method for their severely atrophied mandible (width of 4 mm) was bone grafting with an inverted sandwich technique. 2 split-thickness autogenous calvarial grafts were attained. 4 onlay bone blocks were prepared, placed with an inverted sandwich technique, and fixed with 8 lag screws. 4 implants (Osstem, ⌀4×10 mm) were placed simultaneously, and an immediate post-operation cone beam computed tomography confirmed the proper placement of the grafts and implants. No complications were reported within 4 and 8 months following graft and implant placement. Final impressions for full dental rehabilitation were taken at the 8-month follow-up.

**Conclusion:**

Split-thickness calvarial grafts and simultaneous implant placement seem to be a very efficient and promising approach for major reconstruction of the mandible. However, further studies are recommended.

## 1. Introduction

In severely atrophied jaws, bone augmentation is needed to achieve sufficient bone volume for implant stability and success [1]. Autologous grafts are the gold standard approach, and an extraoral donor site will provide sufficient bone volume for severely atrophied jaws. The iliac crest is the most common extraoral donor site, but it has a high resorption rate (12–60%), leading to late treatment complications. Many complications (pelvic instability, fatigue fracture, etc.) have also been reported [2]. As an alternative withminimal bone resorption, less patient discomfort, and complications, calvarial bone grafting is a viable selection [3]. The method of calvarium bone graft harvesting has been popularized by Tessier [4]. Current studies report the bone stability and the success rate of implants within calvarium grafts to be 94.2% [3].

Implant placement may be done after or with graft placement. Simultaneous implant insertion omits the need for a second intervention, and consolidation of the graft will happen with implant osseointegration. This approach is more technique sensitive, but the healing period for the patient will be reduced [5].

This study reports a successful reconstruction of a severely atrophied mandible with calvarial split bone grafts and simultaneous implant insertion. The grafts were positioned in a special manner to accommodate the patient's needs.

## 2. Case Presentation

A 40-year-old female patient was admitted to our care with the chief complaint of mandibular denture instability. She had been using a tissue-supported denture for 18 years. Her medical history showed no sign of illness, drug use, particular habits, etc. On clinical examination, severe mandibular atrophy was evident, and the attached gingiva was minimal. The mucosa was atrophied, and buccal and lingual vestibules were extremely shallow.

Bone grafts were deemed essential for any sort of rehabilitation, and an implant-supported overdenture was indicated, to prevent future bone resorption. Due to inadequate bone height availability, sandwich osteotomy was not a viable option, and an “inverted” sandwich graft technique was used. Also, calvarial grafts were the best choice for this patient, due to sufficient bone volume availability and less resorption compared to other extraoral sites.

**Figure 1:**
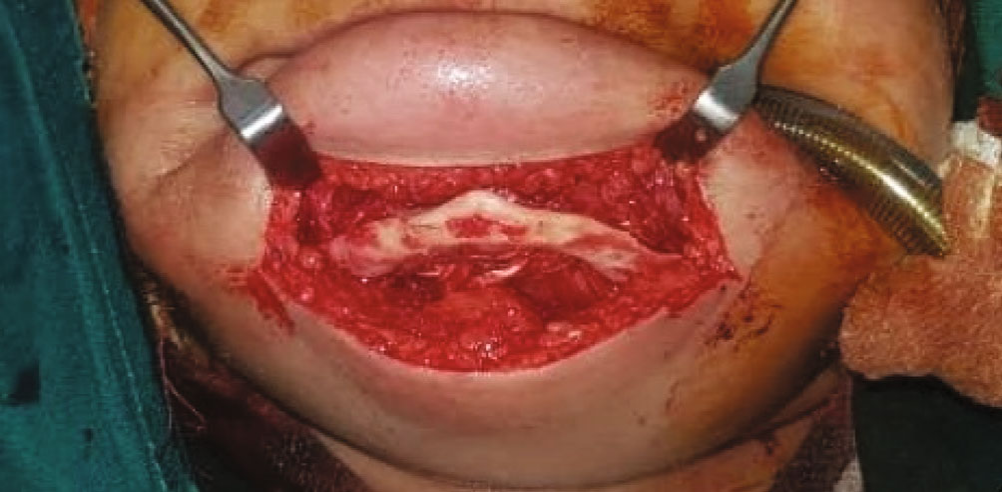
Severely atrophied mandible (mandibular width of 4 mm); genioglossus and geniohyoid muscles were not detached.

The patient's calvarial CT scan showed no defects, and laboratory tests were normal. Surgery was performed with general anesthesia via nasal intubation. An extraoral submandibular incision was made approximately between mandibular premolars. A full-thickness periosteal flap was elevated, and the mandible was fully exposed. The lingual portion was left intact to preserve blood supply (Figure 1).

Decortication was achieved with a fissure bur (*⌀* 2 mm). After a coronal scalp incision and full-thickness flaps, 2 split-thickness grafts (2.5 × 1 cm) were harvested from the outer cortex of the parietal bone. Hemostasis was achieved; flaps were repositioned, fixed, and sutured.

**Figure 2:**
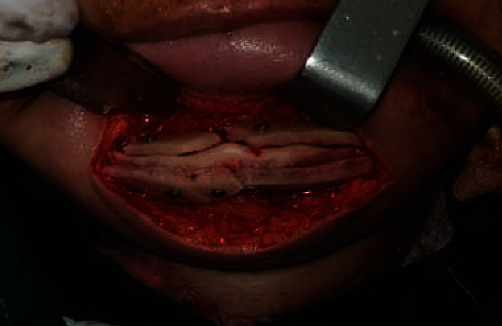
Grafts and lag screws—anterior view.

Bone blocks were positioned, and fixation was done with 8 lag screws (Figure 2). 4 implants (Osstem, ⌀4 × 10 mm) were placed simultaneously. An immediate post-op cone beam computed tomography (CBCT) image was taken, displaying proper implant and screw placement. After surgery, the patient was dismissed within 24 hours.

**Figure 3:**
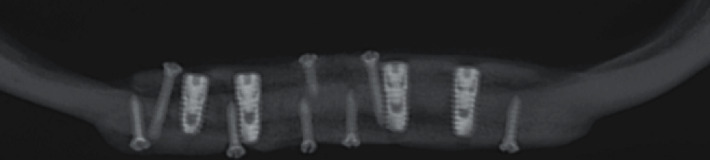
Reconstructed panoramic radiograph of patient's cone beam computed tomography (CBCT)—4-month post-operation.

After 4 months, CBCT imaging showed no sign of complications (Figure 3); thus, implants were exposed and impressions for an overdenture were taken 8 months after implant placement, and the patient was reevaluated for denture instability and peri-implantitis. No complications were observed on clinical examination, and the patient had no complaints.

This report has been written in compliance with the CARE reporting guideline.

## 3. Discussion

In our study, successful reconstruction of a severely atrophied mandible with simultaneous implant insertion was done. Literature suggests that vertical reconstruction of severely atrophied crests (height < 5 mm) be done with block grafts; thus, using an extraoral donor site was inevitable [1, 6]. Also, the inverted sandwich technique (introduced in 2008, very few studies have chosen this method) allowed for a better graft volume distribution [5, 7]. We chose the calvarium as the donor site; as it is well-known for little to no resorption compared to iliac bone grafts, this may be due to different embryonic origins and or differences in microarchitecture [7].

The first documentation of calvarial grafts for severely atrophied ridges reported a 10% resorption rate of calvarial grafts after a year [8]. Chiapasco et al. and Maestre-Ferrín et al. confirm the same findings, reporting resorption rates below 10% [6, 9]. Carinci and co-authors conducted a study comparing iliac and calvarial grafts, reporting a 22% and 10% superiority in bone survival for calvarial grafts, in 10- and 30-month follow-ups [10]. A similar study by Mertens et al. reports resorption rates of 24.16% and 8.44% for iliac and calvarial grafts, respectively [11]. Finally, a review by Smolka reports 97–100% graft success for calvarial grafts, whereas a review by Titsinides et al. reports a resorption rate of 12–60% for iliac crest bone grafts [12].

Even so, many still debate using the calvarium as an extraoral donor site, due to surgical complexity, anatomic considerations (variable calvarial bone thickness, transcortical emissary veins, subcortical vessels, muscle insertions, and aberrant arachnoid plexuses), interoperative (dural tear, superior sagittal sinus involvement, intracranial hemorrhage, etc.), and post-operative (hematoma or seroma of the scalp, alopecia, paraesthesia, irregular bone contour, etc.) complications [13, 14]. To our knowledge, only a study by Gleizal et al. reports 2 incised dural injuries following bicortical graft harvesting of 122 patients. This complication was easily managed and did not lead to cerebral injuries [5, 7–9, 15–20]. In addition, pain and discomfort while walking are common and well-known side-effects of iliac crest graft harvesting. These complications may last up to 2 weeks, whereas donor site complications regarding calvarial grafts are rare and are managed easily [6, 12]. In our study, no complications were encountered, as we harvested split-thickness grafts following a detailed evaluation of the patient's cerebral anatomy. Also, infection was absent in all manipulated areas.

Most studies report uneventful healing and consolidation of the graft, but in some studies, wound dehiscence and/or infection did occur. Smolka and co-authors report wound dehiscence in 3 out of 10 cases and an infection in one case, which lead to 2 partial graft losses [18]. Mertens et al. also report a total of 5 wound dehiscences and partial graft losses [3, 11]. In these studies, the mandible was often accessed via an intraoral incision. We believe this approach, and the volume increase from one dimension might have had an influence on dehiscence occurrence. The submental approach preserves the attached gingiva and implant emergence could occur at, or lingual to the attached gingiva. In all cases, partial graft loss had no effect on final oral rehabilitation success [3, 5, 11, 18].

Simultaneous implant placement is indeed challenging, as it is hard to position the implants properly, especially with a submental approach; but it offers the advantages of concurrent implant osseointegration and graft consolidation, reduced healing period, and reduced number of procedures [5, 7]. Most similar studies until 2011 preferred delayed implant insertion, but recent studies have chosen to place implants simultaneously [1, 12, 13, 19]. In a study by Sakka and Krenkel, simultaneous implant insertion following calvarial grafts in the maxilla resulted in a 94.8% success rate for the implants [13]. A 2021 systematic review and meta-analysis suggests simultaneous implant placement with onlay bone grafts to minimize marginal bone loss and achieve better osseointegration. Also, maximum graft resorption occurs within the first year of graft placement. By placing implants simultaneously and early loading of the graft, the functional stimulus could lead to less resorption and further stability [21]. Lofano et al. approve the same findings and believe that the disuse following latent implant placement could lead to bone resorption, especially with onlay bone grafting [22]. In a study by Yousif and co-authors, delayed insertion was only preferred when immediate placement was not possible. They did not report any significant difference in implant success or survival rates related to the time of implant placement [1].

## 4. Conclusion

Using split-thickness calvarial grafts for major reconstruction of the mandible seems to be a very promising approach. Also, simultaneous implant placement reduced the number of surgical interventions and resulted in no complications. However, surgical expertise and cautionary measurements are definitely advised.

## Data Availability

Upon reasonable request, the corresponding author will provide any data supporting this case with regard to the patient's anonymity.
